# The clinimetric qualities of patient-assessed instruments for measuring chronic ankle instability: A systematic review

**DOI:** 10.1186/1471-2474-8-6

**Published:** 2007-01-18

**Authors:** Christophe Eechaute, Peter Vaes, Lieve Van Aerschot, Sara Asman, William Duquet

**Affiliations:** 1 The Physical Therapy Department, Vrije Universiteit Brussel, Laarbeeklaan 101, B-1090 Brussels, Belgium; 2 The Human Biometry and Biomechanics Department, Vrije Universiteit Brussel, Pleinlaan 2, B-1090 Brussels, Belgium

## Abstract

**Background:**

The assessment of outcomes from the patient's perspective becomes more recognized in health care. Also in patients with chronic ankle instability, the degree of present impairments, disabilities and participation problems should be documented from the perspective of the patient. The decision about which patient-assessed instrument is most appropriate for clinical practice should be based upon systematic reviews. Only rating scales constructed for patients with acute ligament injuries were systematically reviewed in the past. The aim of this study was to review systematically the clinimetric qualities of patient-assessed instruments designed for patients with chronic ankle instability.

**Methods:**

A computerized literature search of Medline, Embase, Cinahl, Web of Science, Sport Discus and the Cochrane Controlled Trial Register was performed to identify eligible instruments. Two reviewers independently evaluated the clinimetric qualities of the selected instruments using a criteria list. The inter-observer reliability of both the selection procedure and the clinimetric evaluation was calculated using modified kappa coefficients.

**Results:**

The inter-observer reliability of the selection procedure was excellent (k = .86). Four instruments met the eligibility criteria: the Ankle Joint Functional Assessment Tool (AJFAT), the Functional Ankle Outcome Score (FAOS), the Foot and Ankle Disability Index (FADI) and the Functional Ankle Ability Measure (FAAM). The inter-observer reliability of the quality assessment was substantial to excellent (k between .64 and .88). Test-retest reliability was demonstrated for the FAOS, the FADI and the FAAM but not for the AJFAT. The FAOS and the FAAM met the criteria for content validity and construct validity. For none of the studied instruments, the internal consistency was sufficiently demonstrated. The presence of floor- and ceiling effects was assessed for the FAOS but ceiling effects were present for all subscales. Responsiveness was demonstrated for the AJFAT, FADI and the FAAM. Only for the FAAM, a minimal clinical important difference (MCID) was presented.

**Conclusion:**

The FADI and the FAAM can be considered as the most appropriate, patient-assessed tools to quantify functional disabilities in patients with chronic ankle instability. The clinimetric qualities of the FAAM need to be further demonstrated in a specific population of patients with chronic ankle instability.

## Background

Lateral ankle sprains are very common sports related ankle injuries. Recurrence rates of ankle sprains of 19% to 70% have been reported [1,2]. Nineteen to 72% of individuals who sustain a lateral ankle sprain have been reported to have residual symptoms and/or develop chronic ankle instability [2-4]. The development of chronic ankle instability has been ascribed to different causes like a delayed muscle reflex of stabilizing lower leg muscles, deficits in lower leg muscle strength, deficits in kinaesthesia, or an impaired postural control [5-8]. Results of the objective measurements in these studies are often conflicting. When evaluating treatments for chronic ankle instability one mainly focusses on the use of clinician-related outcome measures like radiographs [9,10], postural sway [11,12], muscle reaction time [13] or muscle strength [14-16].

The question remains on whose perspective the outcomes should be explored. The importance of the patient's perspective becomes more recognized in health care as it is argued to be the most important criterion for judging the effectiveness of the treatment [17]. Patient-assessed measures provide a feasible and appropriate method for addressing the concerns of the patient in the context of clinical trials [18]. Psychological and psychosocial factors are related to the development of chronic health problems and determine the level of disabilities and participation problems [19]. The International Classification of Functioning, Disability and Health advocates to describe health problems in terms of impairments, disabilities and participation problems. Therefore, in chronic health problems like chronic ankle instability, the degree of present impairments, disabilities, participation problems and a decreased quality of life should be documented from the patients' perspective.

Patient-assessed instruments, like questionnaires, are therefore appropriate tools. But the clinimetric qualities of these instruments should be documented [18]. Haywood et al [20] reviewed multi-item outcome measures for patients with acute ligament injuries of the ankle. Button et al [21] performed a meta-analysis of rating scales in foot and ankle surgery. However in both reviews, the authors were not focussed on studying the clinimetric qualities of patient-assessed outcome measures designed for patients with chronic ankle instability. Decision-making in clinical practice should rely on the results of systematic reviews. Based upon the guidelines for systematic reviews [22], authors should use a criteria list and explicitly describe the operationalization of it. This is important because the decision-making of which instrument is the most appropriate for use in clinical practice, is based upon the rating of the different items of that list. In their review, Button et al [21] did not use a criteria list at all. Haywood et al [20] did not explicitly describe the operationalization of their criteria.

To our knowledge, no available systematic review identified and evaluated the clinimetric properties patient-assessed instruments for chronic ankle instability. Therefore, the purpose and relevance of this review was to systematically search the literature for patient-assessed instruments used in populations with chronic ankle instability and to evaluate the clinimetric qualities of the studied instruments.

## Methods

### Literature search and selection

For the identification of patient-assessed instruments for chronic ankle instability, the following databases were screened until May 2006: Medline from 1966, Cinahl from 1982, Embase from 1994, Sport Discus from 1949, the Cochrane Controlled Trial Register from 1966 and Web of Science from 1972.

To retrieve eligible instruments, Medical Subject Headings (Mesh terms) and key words were combined to describe the patient population and the instrument (table 1). Mesh terms and key words that were used to identify the patient population were: lateral ligament, ankle; ankle joint; ankle*; joint instability; sprain*; unstable; chronic*; multiple and repetitive; functional and recurren*. Mesh terms and key words that were used to identify the instrument were: questionnaire*; scale*; weights and measures; outcome; outcome assessment, score; self-assessment and self-report. Relevant instruments were identified on the basis of title, abstract and the full text version of the retrieved articles. After identification of the instruments, the specific name of eligible instruments was used for an additional computerized search to identify supplementary relevant studies. Also the name of experts in the field was used to identify possibly relevant instruments. Finally, references in articles of selected instruments and reviews were screened [20,21,23,24].

Instruments were included:

- If they were used in articles studying patients with chronic ankle instability.

- If it was exclusively a patient-assessed instrument, containing items related to disabilities (activities), participation problems (participation) or quality of life.

- If one or more clinimetric qualities of the instrument were studied in the retrieved articles.

Instruments were excluded:

- If the instrument was not exclusively patient-assessed or if the instrument contained items not related to impairments, disabilities (activities), participation problems or quality of life.

- If not published in English, French, Dutch, or German.

Based upon these criteria, two reviewers independently selected eligible instruments. Their inter-observer reliability was assessed using the modified kappa coefficient. When disagreement persisted between the two reviewers concerning eligibility of an instrument, a third person (C.E.) was consulted.

### Quality assessment

The clinimetric qualities of the selected instruments were evaluated by means of a checklist used in the review article of Bot et al [25] who studied the clinimetric qualities of shoulder disability questionnaires. This checklist was partly based upon the review criteria of the Scientific Committee of the medical outcome trust [26]. It contains the following items: content validity, readability, reliability, internal consistency, construct validity, floor- and ceiling effects, responsiveness, interpretability, minimal clinical important difference, administration burden and time to administer (table 2). To achieve agreement between the ratings of the reviewers, a pilot testing of the checklist was conducted by evaluating the clinimetric qualities of the Western Ontario Shoulder Instability Index [27] until consensus was reached.

Subsequently, the two reviewers independently evaluated the selected instruments. Items could be rated by "+", " ± ", "-" or "?". An item was rated "+" when sufficient information was available and bias was unlikely. An item was rated " ± " if the available information was unclear or the used method was doubtful. An item was rated "-" if sufficient information was available but the instrument did not met the criteria. An item was rated "?" if no information was available. Modified kappa coefficients were calculated to assess the inter-observer reliability.

If disagreement persisted about the assignment of a score to an item, a third person (C.E.) was consulted to decide about the final rating.

## Results

### Selection

**Figure 1:**
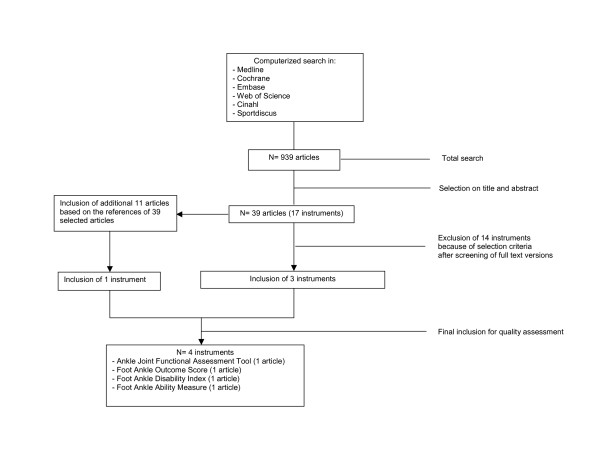
Flow diagram of the selection procedure of the instruments.

The inter-observer reliability of the selection of the instruments was excellent (κ = .86). The search strategy revealed 939 articles (figure 1). Based on the computerized search, 17 instruments were identified comprising 39 articles. After extensively studying the full text version of these 39 articles, 3 instruments met the inclusion criteria: the Ankle Joint Functional Assessment Tool (AJFAT) [28], the Foot and Ankle Disability Index named FADI [29] and the Foot and Ankle Outcome Score (FAOS) [30]. Based upon the references of the 39 articles, the full text version of additional 11 articles was retrieved and studied. This revealed one additional instrument, the Functional Ankle Ability Measure or FAAM [31], which was also included for the quality assessment.

14 instruments were excluded:

- For being a generic health measure (the Short Form Health Survey [32]).

- For containing only "pain related" items (the McGill Pain Questionnaire [33]).

- For not being an exclusively patient-based measure (the Karlsson score, [34], the Kaikkonen scale, [35]; the Zwipp Score, Knop 1999 [36]; the Weber Score, [37]; the Debie Score, [38]).

- For containing no distinct disability, participation or quality of life items (the Good Rating Scale [39]; the Sefton Score[40]; the Keller Score [41], the Subjective Grading Scale [42], the Tegner Score [43], the Subjective Functional Rating Scale [44]).

- Because it contained items not related to impairments, disabilities, participation problems or quality of life (the Brunner Score [45]).

The information regarding the clinimetric qualities of the Ankle Joint Functional Assessment Tool [28], the Foot and Ankle Disability Index [29], the Foot and Ankle Outcome Score [30] and the Functional Ankle Ability Measure [31] was retrieved from the original publications.

### Description of the studied instruments

The Foot and Ankle Outcome Score (FAOS) is a 42-item questionnaire divided into 5 subscales: "pain", "other symptoms", "activities of daily living", "sport and recreation function", "foot and ankle related quality of life". The subscale "pain" contains 9 items, the subscale "other symptoms" 7 items, the subscale "activities of daily living" 17 items, the subscale "sport and recreation function" 5 items and the subscale "foot and ankle related quality of life" 4 items. Each question can be scored on a 5-point Likert scale (from zero to four) and each of the five subscale scores is calculated as the sum of the items included. Raw scores are then transformed to a zero to 100, worst to best score.

The Ankle Joint Functional Assessment Tool (AJFAT) contains 5 impairments (pain, stiffness, stability, strength, "rolling over"), 4 activity related items (walking on uneven ground, cutting when running, jogging and descending stairs) and 1 overall quality item. Each item has 5 answer options. The best total score of the AJFAT is 40 points, the worst possible 0 points.

The Foot and Ankle Disability Index (FADI) is a 34-item questionnaire divided into two subscales: the Foot and Ankle Disability Index and the Foot and Ankle Disability Index Sport. The Foot and Ankle Disability Index contains 4 pain related items and 22 activity related items. The Foot and Ankle Disability Index Sport contains 8 activity related items. Each question can be scored on a 5-point Likert scale (from zero to four). The FADI and the FADI Sport are scored separately. The FADI has a total score of 104 points and the FADI Sport 32 points. The scores of the FADI and FADI Sport are then transformed into percentages.

The FAAM is identical to the FADI except that the "sleeping" item and the 4 "pain related" items of the Foot and Ankle Disability Index are deleted. The Activities of Daily Living subscale of the FAAM (previously called the Foot and Ankle Disability Index) now contains 21 activity related items; the Sports subscale of the FAAM remains exactly the same as the Foot and Ankle Disability Index Sport subscale (8 activity related items). The rating system of the FAAM is identical to the FADI. The lowest potential score of the Activities of Daily Living subscale of the FAAM is 0 points, the highest 84 points. The lowest potential score of the Sports subscale of the FAAM is 0 points, the highest 32 points.

### Quality assessment

The inter-observer reliability for the rating of the items of the checklist was substantial to excellent (κ between .64 and .88). Disagreement between the reviewers existed for the items reliability, construct validity, interpretability and administration burden. The clinimetric qualities are the most extensively documented for the FADI and the FAAM and the least for the AJFAT (see table 3 with the final rating and description of the clinimetric qualities of the studied instruments).

### Clinimetric qualities

A survey of the final rating and the description of the clinimetric qualities of the studied instruments is presented in table 3.

### Content validity

For the AJFAT, no information was available whether patients and experts were involved in the selection and reduction process of items. For the development of the FAOS, patients were asked to rate the relevance and importance of the items from one (not relevant, not important) to three (very relevant, very important). For the FAAM, the refined version of the FADI, both experts and patients were involved in the final item reduction.

### Readability

For none of the studied instruments information on the clarity of the questions for the patients is available.

### Reliability

Test-retest reliability was demonstrated for the FAOS, the FADI and the FAAM. Intra-Class Correlation coefficients (ICCs) for the 5 subscales of the FAOS ranged from .70 to .92. ICCs for the FADI and FADI Sport of the chronically unstable group ranged from .84 to .94. The precision of the measurement (standard error of measurement or SEM) was for the FADI 2,6 points and for the FADI Sport 5,3 points.

ICCs for the Activities of Daily Living subscale and Sport subscale of the FAAM were respectively .89 to .87. The SEMs were respectively 2,1 points and 4,5 points. For the AJFAT, information on test-retest reliability is lacking.

### Internal consistency

Cronbachs' alpha coefficients for the 5 subscales of the FAOS ranged from .88 (for the "pain" subscale) to .97 (for the "sport and recreation" subscale). Cronbachs' alpha coefficients for the Activities of Daily Living subscale and the Sport subscale of the FAAM were respectively .98 and .96. For the AJFAT, information on internal consistency is lacking.

### Floor- and ceiling effects

Ceiling effects, the failure to demonstrate an increased score in patients who clinically improved, were observed for all 5 subscales of the FAOS. 19% of all patients displayed the best possible score for the "foot and ankle related quality of life" scale, 24% for the "symptoms" scale, 30% for the "sport and recreation function" scale, 34% for the "pain" scale and 44% for the "activities of daily living" scale. For the AJFAT, the FADI and the FAAM, no information on floor- and ceiling effects is available.

### Construct validity

The FAOS was correlated to the Karlsson Score; a clinician-assessed scoring scale for ankle instability [34]. Moderate correlation coefficients (Spearman Rho) were found (r = .58 to .67). The ADL and Sport subscales of the FAAM were correlated to the SF-36 physical function subscale and the SF-36 mental function subscale. Strong correlations were found with the SF-36 physical function subscale (r = .84; r = .78), weak correlations were found with the SF-36 mental function subscale (r = .18; r = .11). For the AJFAT, construct validity was not studied in patients with chronic ankle instability.

### Responsiveness

The ability to detect important change of the health status over time was assessed for the AJFAT and the FADI. In the study of Rozzi et al [28] a significant improvement in AJFAT score of trained patients could be observed after 4 weeks of wobble board training but an effect size for the AJFAT score was not presented in their study. Based on their results, we estimated the effect size of the AJFAT to be 2.52.

For both the FADI and the FADI Sport, a significant difference between pre- and post training scores was observed in rehabilitated subjects with chronic ankle instability. Effect sizes for the FADI and the FADI Sport were respectively 0.52 and 0.71.

As well the ADL subscale as the Sport subscale of the FAAM were sensitive to significant changes over time (p < .05). Minimal detectable changes (MDC) were ± 5,7 points for the ADL and ± 12,3 points for the Sport subscale of the FAAM. The Guyatt's responsiveness index for the ADL subscale and the Sport subscale was respectively 2.75 and 1.40 [31].

For the FAOS no information on the responsiveness is available.

### Interpretability

Interpretability was rated positive for the AJFAT, the FADI and the FAAM. In contrast to the AJFAT, the FADI and the FAAM, no detailed information is given about the distribution of the FAOS scores of the 213 patients being studied. Trained patients who demonstrated significant better AJFAT scores also showed a significantly improved postural balance.

Based upon the calculated effect sizes, the FADI Sport seems to be more sensitive to change over time than the FADI. Also, results of the FADI and the FADI Sport scores show that both subscales can discriminate between healthy subjects and subjects with chronic ankle instability.

For the ADL and Sport subscales of the FAAM, means (and standard deviations) and medians (and range of scores) were presented for a subgroup of patients with a variety of foot and ankle problems which was expected to remain stable (n = 79), and for a subgroup of patients which was expected to change (n = 164). In the subgroup of patients who was expected to change over 4 weeks, a significant change in ADL and Sport subscales scores of the FAAM was observed (p < .001).

In the subgroup of patients, which was expected to remain stable, no significant differences in ADL and Sport subscales scores were observed after 4 weeks.

For the ADL and Sport subscales of the FAAM, minimally clinical important differences of respectively 8 and 9 points were presented. For the other instruments, information concerning a minimally clinical important difference was not presented.

Results of the correlation analyses with the SF-36 indicate that the subscales of the FAAM are measures for physical function (r between 0.84 and 0.78) rather than mental function (r between 0.18 and 0.11).

### Time to administer and administration burden

Only for the FAOS, the administration time (7 to 10 minutes) was documented. The final score of the AJFAT is just the result of summing up the different item scores. For the FAOS, the subscale scores are the result of summing up the item scores belonging to that subscale. The raw scores of these subscales are transformed into a 0 to 100 scale.

The scores on the items of the FADI and the FADI Sport are summed up separately and are than transformed into percentages. The scores of the ADL and Sport subscales of the FAAM are calculated in the same manner.

## Discussion

There is no gold standard to evaluate the clinimetric qualities of patient-assessed instruments and hence the criteria list that was used can be disputed. This checklist was chosen for its quality of operationalization.

The inter-observer reliability of the quality assessment of the selected measures was substantial to excellent. Disagreement was mostly caused by reading errors. The third reviewer was not consulted for making a final decision about the rating of the items.

Many rating scales have been used for the evaluation of patients with chronic ankle instability but these are not exclusively patient-assessed and/or do not contain distinct disability, participation or quality of life items. The clinimetric qualities of each studied patient-assessed instrument were described in only one article, despite the systematic and extensive search in literature. As a consequence, patient-assessed instruments are scarcely described in studies related to chronic ankle instability.

Patient-assessed instruments should at least demonstrate validity, reliability and responsiveness before considering them to be useful in clinical practice.

### Content validity

One could expect that the studied instruments would describe more or less the same constructs of chronic ankle instability. However both the FAOS and the AJFAT contain items that refer to impairments, disabilities, participation problems and quality of life while the FADI and the FAAM are mainly developed to document disabilities. Item response theory was used to complete final item reduction of the FAAM and is an important element for studying the content validity of a patient-based instrument. Item reduction should also rely on what patients themselves state not to be important as the degree of importance of an item must primarily be seen from the patients' perspective [17].

### Reliability

There is no strict cut-off point to decide whether an instrument is reliable or not. It has been stated that the magnitude of the correlation coefficient of a measurement tool should at least be .70 when studying groups of patients and exceed .90 when evaluating individuals [18,46]. The FAOS, the FADI and the FAAM met this criterion. However, it must be mentioned that for these instruments items are scored on a Likert scale and scores should be considered as ordinal data. Therefore, it would have been interesting if kappa coefficients have been reported, expressing the degree of agreement between the two test sessions for each single item of the instruments.

### Internal consistency

As well for the FAOS as the FAAM, Cronbach alpha coefficients for the subscales were above .90. This makes it likely that there is some redundancy among items within the subscales of these instruments[18].

### Construct validity

With respect to construct validity the five subscales of the FAOS were correlated to the total Karlsson score. Because one could hypothesize the FAOS to measure the same theoretical construct as the Karlsson Score, it may have been more appropriate to correlate the total scores of these two instruments. Furthermore, correlating the different subscale scores of both instruments would enlighten the construct validity even more.

The results of the correlation analyses of the ADL and Sport subscale with the SF-36 provide evidence of convergent and divergent validity indicating that the FAAM is a measure of physical function rather than mental function.

### Floor and ceiling effects

Floor and ceiling effects were only calculated for the FAOS. According to the quality list used in our study, with the cut-off point set at 15%, all subscales of the FAOS demonstrated ceiling effects. The choice of cut-off point remains arbitrary. For instance, Barber-Westin et al (1999) [47] studied the presence of floor- or ceiling effects of the Cincinatti knee rating system using a cut-off point set at 33%. The observation of ceiling effects may also be specific for the patient population being studied [18]. The patients that were studied had undergone an anatomical reconstruction of the lateral ankle ligaments on average 12 years prior to the study (Roos et al [30]). It is probable that many of them no longer had ankle problems, which may explain the observation of ceiling effects. Moreover, 34% of the same patients also obtained the best possible Karlsson Score. The high percentage of ceiling effects in the FAOS "pain" subscale and FAOS "activities of daily living" subscale may compromise the validity of these subscales.

The subjects with chronic ankle instability that were studied by Hale and Hertel [29] have at baseline substantially high FADI and FADI Sport scores. This indicates that these subjects do not demonstrate much difficulties and are functioning on high-level ability. The absence of ceiling effects for the FADI and the FADI Sport should be established.

In the study of Martin et al [31], highest and lowest possible scores were observed in both the ADL subscale and the Sport subscale of the FAAM. This may indicate the presence of floor and ceiling effects.

### Responsiveness

To establish responsiveness, several estimates (like effect sizes or standardized response means) can be calculated which permits comparison of the sensitivity to change between several instruments. The observed difference in effect size between the FADI (ES = 0.52) or the FADI Sport (ES = 0.71), representing a medium size of change [18], and the AJFAT score (ES = 2.52), representing a large size of change, may indicate that the AJFAT is a more responsive measure.

In the study of Hale and Hertel [29], the FADI Sport is more responsive than the FADI. However, in the study of Martin et al [31], the Sport subscale of the FAAM, although identical to the FADI Sport, seems to be less responsive than the ADL subscale. These conflicting results may be explained by the difference in patients being studied. In the study of Martin et al [31], patients with a variety of foot and ankle problems were evaluated, while Hale and Hertel [29]. studied subjects with chronic ankle instability. The difference in the size of minimal detectable change between the FADI Sport (6,39 points) and the Sport subscale of the FAAM (12,3 points) also may explain these contrasting findings.

From the MCIDs of the ADL subscale (8 points) and Sport subscale (9 points) of the FAAM, one can be 95% confident that a patient would wrightfully consider his or herself as having improved or deteriorated when the change of score exceeds 8 points (ADL subscale) or 9 points (Sport subscale).

The FAAM received the most positive ratings for its clinimetric evaluation. However, one must take into account that these clinimetric properties are established in a patient population with a variety of foot and ankle problems. The clinimetric properties of the FAAM should also be further demonstrated in a specific population of patients with chronic ankle instability.

## Conclusion

A systematic computerized literature search of 6 databases revealed 4 patient-assessed instruments for measuring chronic ankle instability: the Ankle Joint Functional Assessment Tool, the Foot and Ankle Disability Index, the Foot and Ankle Outcome Score and the Foot and Ankle Ability Measure. The FADI and the FAAM can be considered as the most appropriate, patient-assessed tools to quantify functional disabilities in patients with chronic ankle instability. The clinimetric qualities of the FAAM need to be further demonstrated in a specific population of patients with chronic ankle instability.

## Competing interests

The author(s) declare that they have no competing interests.

## Authors' contributions

CE contributed to the concept and design of the manuscript, to the acquisition, analysis and interpretation of the data and drafted the manuscript. PV contributed to the concept and design of the manuscript and participated in the interpretation of the data. LVA contributed to the concept and design of the manuscript, to the acquisition and interpretation of the data and drafted the manuscript. SA contributed to the concept and design of the manuscript, to the acquisition and interpretation of the data and drafted the manuscript. WD contributed to the acquisition, analysis and the interpretation of the data. All authors read and approved the final manuscript.

## Pre-publication history

The pre-publication history for this paper can be accessed here:



## Figures and Tables

**Table 1:** Literature search in the Medline database

#27	Search #18 AND #26	257^$^
#26	Search #19 OR #20 OR #21 OR #22 OR #23 OR #24 OR #25	989004
#25	Search outcome [TIAB]	245359
#24	Search score [TIAB]	94893
#23	Search self-assessment [TIAB]	2896
#22	Search self-report [TIAB]	11048
#21	Search measure* [TIAB]	158645
#20	Search questionnaire [TIAB]	95367
#19	Search "Questionnaires"[MeSH] OR "Weights and Measures"[MeSH] OR "Outcome Assessment"(Health Care)"[MeSH] OR "Treatment Outcome"[MeSH]	466731
#18	Search #4 AND #10 AND #17	895
#17	Search #11 OR #12 OR #13 OR #14 OR #15 OR #16	1429234
#16	Search multiple [TIAB]	357243
#15	Search repetitive [TIAB]	27785
#14	Search functional* [TIAB]	425161
#13	Search recurren* [TIAB]	194753
#12	Search chronic* [TIAB]	473604
#11	Search "Recurrence"[MeSH]	100803
#10	Search #5 OR #6 OR #7 OR #8 OR #9	83178
#9	Search inversion [TIAB]	13941
#8	Search instability [TIAB]	34623
#7	Search sprain* [TIAB]	2071
#6	Search unstable [TIAB]	31862
#5	Search "Sprains and Strains"[MeSH:NoExp] OR "Joint Instability"[MeSH]	9501
#4	Search #1 OR #2 OR #3	19963
#3	Search ankle* [TIAB]	17290
#2	Search "Lateral Ligament, Ankle"[MeSH]	136
#1	Search "Ankle Joint"[MeSH]	5761

* = abbreviation, Mesh = Medical Subject Heading; [TIAB] = key word in title or abstract; ^$ ^= final number of hits.

**Table 2:** Checklist for rating the clinimetric qualities of self-assessment instruments.

Clinimetric quality	Definition	Criteria to rate the clinimetric quality
Content validity	The extent to which the domain of interest is comprehensively sampled by the items in the measure	1) Patients and experts were involved during item selection/reduction 2) Patients were consulted for reading and comprehension Rating: + patients and experts were involved ± only patients were involved - no patient involvement ? no information found on content validity
Readability	The questionnaire is understandable for all patients	Rating: + reading was tested and result was good - inadequate readability ? no information about readibility
Reliability	The extent to which the same results are obtained on repeated administrations of the same measure when no change in physical functioning has occurred (reliability) or the extent to how precise the scores are on repeated measurements (agreement)	1) Correlation coefficient (r > .70); limits of agreement, kappa or standard error of measurement are presented Rating: + adequate design, method and r > .70 ± doubtful method used - inadequate reliability or agreement ? no information found on reliability or agreement
Internal consistency	The extent to which items in a subscale are inter-correlated; a measure of the homogeneity of the subscale	1) Factor analysis was applied in order to provide the dimensionality of the measure 2) Cronbach's alpha between .70 an .90 for each subscale Rating: + adequate design, factor analysis; alpha: .70 – .90 ± doubtful method used - inadequate internal consistency ? no information found on internal consistency
Construct validity	The extent to which scores relate to other measures in a manner that is consistent with theoretically derived hypothesis concerning the domains that are measured	1) Hypotheses were formulated 2) Results were acceptable in accordance with the hypotheses Rating: + adequate design, results in accordance with the hypotheses ± doubtful method used - inadequate construct validity ? no information found on construct validity
Floor-ceiling effects	The measure fails to demonstrate a worse score in patients who were clinically deteriorated and/or an improved score in patients who clinically improved	1) Descriptive statistics of the distribution of scores were presented 2) 15% of the respondents achieved the highest or lowest possible score Rating: + no floor- and ceiling effects - > 15% in extremities ? no information found on floor-ceiling effects
Responsiveness	The ability to detect important change over time in the concept being measured	1) Hypotheses were formulated and results were in agreement 2) An adequate measure was used (effect size, standard response mean or comparison with external standard) Rating: + adequate design, method and result ± doubtful method used - inadequate responsiveness ? no information found on responsiveness
Interpretability	The degree to which one can assign qualitative meaning to quantitative scores	Authors provided information on the interpretation of scores: 1) Presentation of means and standard deviations of scores 2) Comparative data in relevant subgroups 3) Information on the relationship of scores to well-known functional measures or clinical diagnosis 4) Information on the association between change in scores and patients global ratings of the magnitude of change they have experienced Rating: + 2 or more types of information was presented ± doubtful method used or doubtful description ? no information found on interpretability
Minimally clinical important difference (MCID)	The smallest difference in scores in the domain of interest which patients perceive as beneficial and would mandate a change in patients' management	Information is provided about what difference in score would be clinically meaningful Rating: + minimally clinical important difference presented - no minimally clinical important difference presented
Time to administer	Time needed to complete the measure	Rating: + less than 10 minutes - more than 10 minutes ? no information
Administration burden	Ease of method used to calculate the questionnaire's score	Rating: + easy: summing up the items ± moderate: visual analogue score or simple formula - difficult: complex formula ? no information found on rating method

**Table 3:** Final rating and description of the clinimetric properties of the studied instruments.

Clinimetric property	Item rating	AJFAT^1^	Item rating	FAOS^2^	Item rating	FADI^3^	Item rating	FAAM^4^
Content validity	?	No information	±	Item selection and reduction by patients (n = 213) Experts: not involved	+	Experts and patients were involved in item generation and reduction	+	Experts and patients were involved in item generation and reduction

Readability	?	No information	?	No information	?	No information	?	No information

Reliability	?	No information	+	subscale pain: r_s _= .96; subscale symptoms: r_s _= .89; subscale ADL: r_s _= .85; subscale sports: r_s _= .92; subscale quality of life: r_s _= .92	+	FADI involved ankles: ICC = .89, SEM = 2.61; FADI uninvolved ankles: ICC = .85, SEM = 0.82 FADI Sport involved ankles: ICC = .84, SEM = 5.32; FADI Sport uninvolved ankles: ICC = .94, SEM = 0.99	+	ADL subscale: ICC = .89; SEM = 2,1 points Sport subscale: ICC = .87; SEM = 4,5 points

Internal consistency	?	No information	-	subscale pain: α = .94; subscale symptoms: α = .88; subscale ADL: α = .97; subscale sports: α = .94; subscale "quality of life": α = .92	?	No information	-	Cronbach alpha for ADL subscale: α = .96 in stable group (n = 79); in changed group: α = .98 (n = 164) Cronbach alpha for Sport subscale from a combined sample: α = .98

Construct validity	?	No information	+	Correlation of the 5 subscales to the KS: r = between .58 – .67	?	No information	+	Correlation with SF-36 physical component: ADL subscale: r = .84; Sport subscale: r = .78 Correlation with SF-36 mental function: ADL subscale: r = .18; Sport subscale: r = .11

Responsiveness	+	Significant difference after 4 weeks of balance training: pre experimental score = 17.11 (± 3.44) post experimental score = 25.78 (± 3.8); ES = 2.52 (n = 13 patients)	?	No information	+	FADI; significant difference after 6 weeks of training: pre training score =87.1% (± 12,1) post training score = 94.4% (± 6,1) ES = 0.52 (n =16 subjects) FADI Sport; significant difference after 6 weeks of training: pre training score = 78.4% (± 12,9)post training score = 89.5% (± 11,3; ES = 0.71 (n = 16 subjects) MDC FADI = ± 4,48 points; MDC FADI Sport = ± 6,39 points	+	Significant change in ADL subscale percentage score in group expected tochange after 4 weeks: pre = 58,0% (± 24,8); post = 74,9% (± 20,0)compared to the group expected to remain stable: pre = 91,5% (± 13,6);post = 92,6% (± 13,2) (p < .001) Significant change in Sport subscale percentage score in group expected to change after 4 weeks: pre = 25,2% (± 26,7); post = 43,9% (± 30,0) compared to the group expected to remain stable: pre = 78,6% (± 23,8); post = 81,9% (± 23,3) (p < .001)

Responsiveness								GRI of ADL subscale = 2.75; Sport subscale = 1.40 MDC of ADL subscale = ± 5.7 points; Sport subscale = ± 12.3 points

Interpretability	+	Means and sds. of AJFAT scores were presented. A significant improvement on the AJFAT score was accompanied with significant improvement on postural balance in trained patients	?	No information	+	Moderate correlation (r = .64) between FADI scores and FADI Sport scores in involved ankles of the chronically unstable ankle group Involved ankles of the chronically unstable ankle group have significantly worse scores on FADI- and FADI Sport than healthy controls.	+	Means and medians of ADL subscale scores and Sport subscale scores were presented No significant change in scores of group expected to remain stable; significant change in scores of group expected to change Strong correlations between ADL subscale scores and Sport subscale scores and SF-36 physical component Weak correlations between ADL subscale scores and Sport subscale scores and SF-36 mental component Patients who perceived themselves as being improved showed an increased score of respectively 8 (ADL subscale) and 9 points (Sport subscale)

MCID	-	No MCID presented	-	No MCID presented	-	No MCID presented	+	MCID of ADL subscale = 8 points; Sports subscale = 9 points

Administration burden	+	Total score is the result of summing up individual items	±	Raw scores are transformed into a zero to 100 total score	±	Total score is transformed into percentages	±	Total score is transformed into percentages

Administration time	?	No information	+	Less than 10 minutes	?	No information	?	No information

AJFAT = Ankle Joint Functional Assessment Tool; FAOS = Foot and Ankle Outcome Score; FADI = the Foot and Ankle Disability Index; FAAM = Foot and Ankle Ability Measure; 1 = Rozzi et al, 1999; 2 = Roos et al, 2001; 3 = Hale and Hertel, 2005; 4 = Martin et al, 2005; MCID = minimal clinical important difference; ADL = Activities of Daily living
